# Influenza Pandemics in Singapore, a Tropical, Globally Connected City

**DOI:** 10.3201/eid1307.061313

**Published:** 2007-07

**Authors:** Vernon J. Lee, Mark I. Chen, Siew Pang Chan, Chia Siong Wong, Jeffery Cutter, Kee Tai Goh, Paul Anath Tambyah

**Affiliations:** * Tan Tock Seng Hospital, Singapore; † National Healthcare Group, Singapore; ‡ Ministry of Defence, Singapore; § Ministry of Health, Singapore; ¶ National University of Singapore, Singapore; # National University Hospital, Singapore

**Keywords:** Influenza, pandemic, history, effect, policy, prevention, historical review

## Abstract

Tropical cities such as Singapore do not have well-defined influenza seasons but have not been spared from influenza pandemics. The 1918 epidemic in Singapore, which was then already a major global trading hub, occurred in 2 waves, June–July, and October–November, and resulted in >2,870 deaths. The excess mortality rate was higher than that for industrialized nations in the Northern Hemisphere but lower than that for less industrialized countries in Asia and Africa. The 1957 epidemic occurred in May and resulted in widespread illness. The 1968 epidemic occurred in August and lasted a few weeks, again with widespread illness. Tropical cities may be affected early in a pandemic and have higher mortality rates. With the increase in travel and trade, a future pandemic may reach a globally connected city early and spread worldwide. Preparedness and surveillance plans must be developed to include the megacities of the tropical world.

## Abstract 2

Pandemic preparedness plans must include cities that are global trading hubs.

Influenza has had a substantial effect worldwide. The 3 influenza pandemics of the 20th century (1918–9, Spanish Flu; 1957–8, Asian Flu; and 1968, Hong Kong Flu) resulted in 40 million, 2 million, and 1 million deaths, respectively (*1*,*2*). Their social, cultural, and economic effect has been best described in North America and Western Europe (*3*).

Although tropical countries such as Singapore do not have as well-defined influenza seasons as temperate regions, they are not spared from the effects of influenza (*4*). Each year, 20% of Singapore’s population is estimated to be clinically infected from seasonal influenza (*5*). Deaths caused by influenza in Singapore over the past decade were ≈14.8 per 100,000 person-years, which is comparable to deaths caused by this disease in the temperate United States and subtropical Hong Kong Special Administrative Region, People’s Republic of China (*6*). However, the effect of pandemic influenza in tropical cities has not been well described. This study aims to describe the effect of these pandemics on Singapore, a global trading city throughout the 20th century. The lessons learned from the effect and management of previous pandemics may have implications for pandemic planning in tropical global trading cities.

## Methods

To determine the effect of influenza on mortality rates during the pandemic years, we obtained monthly mortality rate data from various official sources in Singapore. For the years surrounding the 1918 pandemic, data were obtained from the Annual Departmental Reports of the Straits Settlements (the British colonies that included Singapore, Penang, Malacca, and Labuan; the last 3 are now part of Malaysia), and from the Registry of Births and Deaths, Singapore. For the years surrounding the 1957 and 1968 pandemics, data were obtained from the Registry of Births and Deaths, Singapore. These were the only official government departments responsible in the respective years for the collection and verification of these statistics.

Because tropical countries do not have well-defined influenza seasons, methods for the analysis of excess deaths in temperate countries such as that used by Serfling et al. (*7*) may not be appropriate because the assumption of influenza seasons in distinct, regular waves may not be valid. We have thus elected to use direct statistical analysis of data for the 2 years before and after the pandemic year to form a regression line with 95% confidence intervals. Deaths for each month were then compared with the regression line. Months for which the mortality rate exceeded the 95% confidence intervals were considered as those with excess deaths, with the excess represented as the actual mortality rate minus the predicted mortality rate.

To provide another perspective of possible excess deaths for comparison, we used another method described by Murray et al. for 1918, with a simpler equation to estimate excess deaths (*8*). For 1918, death rates during the 3-year pandemic window were compared with those in surrounding years, i.e., the average mortality rate for the 3 years before and after were subtracted from the mortality rate during the 3-year pandemic window.

In addition to statistical analyses, we conducted a detailed search of peer-reviewed journal articles, government reports, and press articles for the 3 pandemics. Search results provided comparisons of the mortality rates in other countries and an overview of the public health issues and interventions conducted in Singapore and how they compared with those of other countries and current recommendations.

## Results

### The 1918 Pandemic in Singapore

The 1918 Straits Settlements Annual Report described an influenza epidemic in June and July that was relatively mild, with a high illness rate but a low mortality rate, that peaked during the week ending July 6 (*9*). A second intense wave occurred in October and November, leading to frequent pneumonia and a high mortality rate. It peaked during the week ending October 26, with 97.6 deaths per million population (*10*).

The 1918 Annual Report indicated 844 recorded influenza deaths. However, the Straits Settlements’ overall annual mortality rate was “43.85 per thousand in 1918 when the influenza epidemic struck the country” (*10*). This is in contrast to the immediate prepandemic and postpandemic years from 1915 to 1921, when mortality rates ranged from 29/1,000 to 37/1,000 population. The excess mortality rate within the Straits Settlements in 1918 was therefore 11.3/1,000 (9,435/827,719).

**Figure 1:**
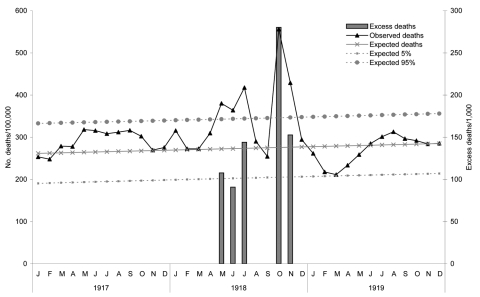
All-cause monthly mortality rates, Singapore, 1917–1919.

Figure 1 shows that the excess mortality rate of the epidemic in Singapore alone, as calculated by our method, was 7.76 per 1,000 (2,870/369,800) during May–June and October–November 1918. Using the formula of Murray et al. (*8*), we calculated the excess mortality rate for Singapore during the pandemic years of 1918–20 to be 1.80% (18/1,000, or 6,656 deaths).

The excess mortality rate for Singapore during the 1918 pandemic years was higher than rates for most industrialized countries such as the United States and those for western Europe (Table), but lower than rates for African and Asian countries such Kenya, South Africa, India, and the Philippines. The excess mortality rate of 1.80% for Singapore was higher than the global average rate of 1.06% and higher than the rate for other Asian countries such as Taiwan (1.44%) (*8*).

**Table:** Estimated deaths and mortality rates due to influenza during the 1918–1920 influenza pandemic

Country	No. deaths (in 1,000s)	Mortality rate (per 1,000), %	References
United States	402–675	3.9–6.5	(*8*,*11*–*13*)
Canada	50.0–51.0	6.1–6.3	(*8*,*12*)
Denmark	6.02–12.4	2.0–4.1	(*8*,*12*)
England	116–200	3.4–5.8	(*8*,*12*)
Spain	257–311	12.3–14.9	(*8*,*12*)
Portugal	59.0–159	9.8–26.4	(*8*,*12*)
India	185	6.1–43.9	(*8*,*12*)
Japan	368–517	6.7–9.4	(*8*,*12*)
Ceylon (Sri Lanka)	51.0–91.6	10.0–17.9	(*8*,*12*,*14*)
Taiwan	25.4–52.8	6.9–14.4	(*8*,*12*)
The Philippines	81.0–288	8.0–28.4	(*8*,*12*)
Argentina	10.2–46.0	1.2–5.4	(*8*,*12*)
Australia	14.5–15.4	2.7–2.9	(*8*,*12*)
Kenya	104–150	40–57.8	(*8*)
South Africa	300	44.3	(*8*)
British Honduras (Belize)	1.01–2.00	2.3–4.6	(*15*)
Trinidad and Tobago	0.30–1.00	0.1–0.2	(*15*)
Singapore	2.87–6.66	7.8–18.0	This report

To reduce the effect of the pandemic, the government used available evidence to institute a series of preventive measures. The government and physicians advised infected persons to isolate themselves and seek treatment, to disinfect the floors of public premises daily, and, during the second wave of the outbreak, to avoid crowded places (*16*). Suggestions were also made to restrict or prohibit visiting of hospitalized patients, and schools were closed for a week at the peak of the second wave (*16*). Recommended prophylactic measures included reducing the amount of fatigue and maximizing ventilation.

By the end of November 1918, the epidemic was over in Singapore, although the media still reported the disease in Indonesia, New Zealand, South Africa, Japan, and other regions. There were no local reports or evidence of a third wave similar to that in temperate countries in early 1919 (*17*).

### The 1957 Pandemic in Singapore

**Figure 2:**
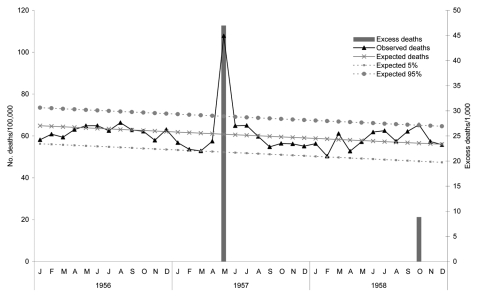
All-cause monthly mortality rates, Singapore, 1956–1958.

The media declared the 1957 pandemic as the “worst ever in colony [Singapore] history” (*18*). The outbreak was first recognized at the end of April and early May and was purported to have spread through Hong Kong from its origins in northern Asia (*19*). By May 5, the outbreak had become an epidemic, reaching its peak in mid-May and tapering off by the end of the month (*20*). In May, 77,211 (47.6%) of 162,093 patients who came to government and city council clinics were treated for influenza; 326 required hospital admission, and 28 deaths from influenza were recorded (22 from pneumonia and 6 from cardiac complications) (*20*). On the basis of monthly mortality rate reports (Figure 2), an excess mortality rate of 0.47/1,000 occurred in May 1957. This represented 680 deaths in a population of 1,445,900. There was another small peak of excess deaths in October 1958, although this was only slightly above the baseline value.

During the 1957 epidemic, the government focused on public health measures, including closure of schools for almost 2 weeks because of illness and absenteeism. The public was advised to keep away from crowded places (*20*), and the slogan “no movement of persons – no spread of influenza” was professed (*21*). At healthcare facilities, elective surgery was minimized to release staff to manage the epidemic. School health clinics, maternal and child health clinics, and voluntary clinics were set up as influenza treatment centers (*20*). Although no port quarantine measures were required by law, the airport health officer checked outward-bound passengers for airlines upon request. Similarly, 1 shipping line screened all passengers boarding their ships, and those who failed screening were denied embarkation (*20*).

### The 1968 Pandemic in Singapore

The 1968 pandemic was the mildest of the 3 pandemics; the epidemic in Singapore occurred in early August and lasted for a few weeks. The virus was believed to have spread from a major outbreak in Hong Kong (*22*).

**Figure 3:**
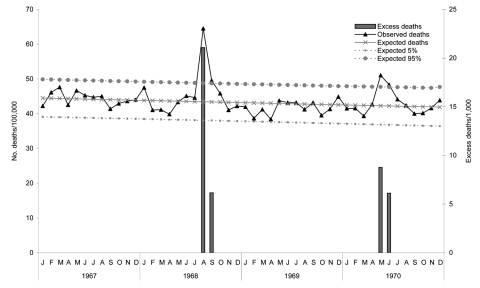
All-cause monthly mortality rates, Singapore, 1967–1970.

The outbreak in Singapore peaked August 16–25. Attendance at outpatient dispensaries increased over a 2-week period, and at the peak daily attendance increased 65% from 6,052 to 9,966 (*23*). On the basis of monthly mortality rates (Figure 3), the excess mortality rate was 0.27/1,000 (543/2,012,000) during August and September 1968. Excess deaths peaked again in May and June 1970, which mirrored a possible second pandemic wave, as reported worldwide in 1969–70, although the lower second wave excess mortality rate was similar to rates in the Americas and different from rates in Europe and Asia (*24*). The excess mortality rate for 1970 was 0.15/1,000 (309/2,074,500).

The 1968 epidemic caused substantial illness and absenteeism from work. However, because of the relatively mild and short epidemic, no substantial measures were adopted. The Ministries of Education and Health considered the closure of schools but decided against it because of the waning of the epidemic (*25*).

## Discussion

Excess mortality rates vary according to the method used for calculation and sources of data, facts that reiterate the difficulty of conducting historical estimates. Nevertheless, the estimated number of influenza deaths (2,870–6,656) in Singapore in 1918 exceeded the official report of 844 influenza deaths. The 1918 Annual Report admitted that the latter number poorly represented actual deaths, which it estimated more accurately at 3,500 (*9*). The 1921 Annual Report added that many deaths reported as pneumonia were due primarily to influenza (*10*). This showed that tropical Singapore had mortality rates comparable to or exceeding those of temperate regions (Table). Similarly, the calculated excess deaths of 680 in 1957 exceeded the 28 recorded influenza deaths.

The excess mortality rate for Singapore (Table) supports the hypothesis that income levels and development were negatively correlated with influenza mortality rates (*8*,*26*) because Singapore was less industrialized than many industrialized Western cities and nations in the early 20th century. However, Singapore, as a main trading city, was relatively more industrialized with a proportionately smaller rural population, and thus had lower mortality rates than did neighboring countries such as India and the Philippines (Table). Even in Singapore, attack rates were lower for Europeans and Asians with higher socioeconomic status (6.0%–20.4%) than for persons with a lower socioeconomic status (29.0%–29.8%), which suggested that socioeconomic status had a possible role in disease transmission (*19*,*27*). Another possible explanation is that those who were more educated were also more receptive to public health messages, which reduced disease transmission.

Using the formula of Murray et al. for the 1918–20 pandemic, we determined that the excess mortality rate for Singapore was higher than the global average rate. Because the Singapore epidemic occurred early in the global pandemic, this finding corroborates the suggestion that early epidemic centers experienced higher mortality rates (*8*). This is also evident when one compares the mortality rates for tropical countries such as Ceylon (present day Sri Lanka) and Singapore with rates for tropical Caribbean islands (Table). During the second wave of the pandemic in Spain in October 1918, Asian nations such as Ceylon and Singapore also reported similar epidemic recrudescence in early October (*28*). By the end of the second wave of the pandemic in Singapore, there were still reports of influenza in Malaysia, Indonesia, New Zealand, and Japan (*9*,*29*). This finding also suggests that nations are at high risk of acquiring early infection and could act as sentinels for the next pandemic.

The effect of all 3 pandemics was felt across Singapore. However, reported overall mortality rates of 43.85/1,000 in 1918 were comparable to “46.46 per thousand in 1911, a very malarious year” (*10*). Deaths during the first epidemic wave were initially attributed to malaria (*30*). The 1918 pandemic also had a variable effect in US possessions in tropical regions (*3*). The early effect from the 1918 Singapore epidemic may not have been noticeable because of the nonseasonal nature of influenza in the tropics (*4*) or because of the high background mortality rates from infectious diseases and other causes in Singapore. Although excess deaths in 1918 were substantially higher than excess deaths in 1957, the relative change in mortality rates was similar; peak monthly mortality rates were twice baseline mortality rates for both periods (Figures 1, 2). The 1918 baseline mortality rate was 4× higher than the 1957 rate, and the decrease in the baseline mortality rate was largely due to improved socioeconomic conditions and control of infectious diseases such as malaria. With the low baseline mortality rate for modern cities, the effect of a pandemic, however mild, may be noticed (the Singapore media declared the 1957 pandemic as the worst). Although studies suggest that pandemic mortality rates will be higher for industrialized countries (*8*,*26*), if a pandemic were to first appear in less industrialized regions with high baseline mortality rates, the pandemic might be missed or dismissed as yet another spike of endemic infectious diseases during the initial epidemic phase until deaths increased.

Apart from illness and death, subpopulations were also severely affected by the pandemics. In 1957, the closure of 670 schools affected 262,000 students who required alternative care and education. Commercial firms reported staff absenteeism of 10%–30% (*31*). Clinics were frequently overwhelmed, and available healthcare workers were recalled to cope with the increase in influenza patients. However, healthcare workers were at high risk for infection (*14*,*31*). In 1918, 12 (63%) of 19 nurses at the Singapore General Hospital were concurrently ill (*32*); in 1957, 25% of the nursing staff in Taiping (a Malaysian town) were ill (*33*). Healthcare workers were stressed as they coped with personal illness and increased numbers of patients.

Although more is now known about influenza pathology and epidemiology, in 1918, influenza was correctly reported as being highly infectious and spread by breathing, coughing, and spitting, and having an incubation period “from a few hours to three days” (*34*). Even with the knowledge gap, measures such as respiratory hygiene, social distancing, and disinfection were promoted (*16*,*19*). In the recent World Health Organization recommendations for pandemic influenza, respiratory hygiene has been encouraged as a routine preventive measure (*35*). Social distancing and disinfection may also be considered to reduce its effect, depending on severity and transmission of disease, to reduce its effect, although definitive evidence is lacking (*35*). The effect of school closures remains unclear. Ferguson et al. suggested that closure of schools does not substantially reduce overall attack rates but does reduce peak attack rates (*36*). Germann et al. suggested that school closures may be effective if conducted early in pandemics with low reproductive numbers (low *R_o_* values) (*37*). However, interventions such as travel restrictions and border controls have been shown to be not feasible (*36*). Although some of these measures may reduce illness and death, they have to be weighed against productivity losses and socioeconomic effects of the interventions.

With the increase in travel and trade, a future pandemic may reach a globally connected city before preparedness plans can be fully activated. The 1918 pandemic is thought to have originated early in the year and had spread to Singapore by June. Another globally connected city, New York City, also showed an early wave in February–April 1918 (*38*). The 1957 and 1968 epidemics arrived weeks after their suspected origins in northern Asia because of travel from Hong Kong, another globally connected city (*21*,*23*). These type of cities are also the focal point of spread, as shown by the spread of influenza from Singapore to India in 1957 (*39*).

Mortality rates suggest that the 1918 epidemic in Singapore may have occurred in May, which is earlier than in official reports (Figure 1). This finding suggests the possibility of late recognition. Delayed recognition must be considered even in this modern age. In 2003, the severe acute respiratory syndrome (SARS) epidemic reached Singapore within weeks of its appearance in the southern part of the People’s Republic of China but remained undetected. Two of the 20th-century influenza pandemics and the SARS epidemic are believed to have originated from farms in eastern Asia. SARS was first detected in Foshan, quickly spread to Guangzhou City (a major regional trading hub), to Hong Kong, and then to the rest of the world. A global surveillance effort is therefore critical to enable prompt activation of pandemic plans. This effort should include frontline surveillance of farms in eastern Asia and secondary surveillance of major Asian cities.

Trading hubs may be affected early in the course of a pandemic and show higher mortality rates. The megacities of Asia, Africa, and Latin America are now extensively involved in global trade and travel networks and are more likely to be affected by a pandemic. However, influenza is a difficult surveillance target, with an accuracy of clinical diagnosis in 1968 of only ≈66% (*40*). A good laboratory surveillance network in major cities is therefore critical to enable accurate diagnosis and virus identification.

This study has some limitations. Mortality rate data in Singapore, although of good quality because of the small size of the country, were available only from limited sources. We have attempted to use estimates from other government agencies such as the health department and ministry of health. Weekly data would have provided better information, but quality data were available in the press only for certain weeks, which we have presented.

Globally connected cities will be especially vulnerable to a future pandemic, and preparedness plans must be developed to include the megacities of the tropical world. The 20th-century pandemics swept through Singapore within 4 weeks; future plans must include such a possibility over a similarly short duration. Public health measures such as surveillance and preparedness plans must be formulated to slow the spread of a pandemic and mitigate its effects.
